# Assessment of contamination using an ATP bioluminescence assay on doorknobs in a university-affiliated hospital in Japan

**DOI:** 10.1186/s13104-015-1305-7

**Published:** 2015-08-14

**Authors:** Naoko Kajigaya, Yoneji Hirose, Shinta Koike, Tomohiro Fujita, Norio Yokota, Satsuki Hata, Makoto Ikenaga, Noritada Kobayashi, Takashi Takahashi

**Affiliations:** Department of Infection Control and Prevention, Kitasato University Medical Center, 6-100 Arai, Kitamoto, Saitama, 364-8501 Japan; Department of Clinical Laboratory, Kitasato University Medical Center, 6-100 Arai, Kitamoto, Saitama, 364-8501 Japan; Laboratory of Infectious Diseases, Graduate School of Infection Control Sciences, Kitasato University, 5-9-1 Shirokane, Minato-ku, Tokyo, 108-8641 Japan

**Keywords:** Doorknob, Contamination, ATP bioluminescence assay, Stamp culture, University hospital

## Abstract

**Background:**

Doorknobs are inevitable points of hand contact. We monitored doorknob contamination in a university hospital using an ATP bioluminescence assay and stamp agar method. We selected grip-, lever-, push-, insert-, and two-pull-type doorknobs in staff lavatories and break rooms, a linen closet, dirty utility rooms, a newborn care unit, clinical lavatories and examination rooms, dressing rooms for radiological tests, and lavatories for health examination, as monitoring points in wards and clinics. Sequential monitoring with an ATP assay (six times) and culture (once) were performed at the same time of day in autumn, winter, and summer. We provided contamination data to appropriate healthcare providers and housekeepers, and queried the staff regarding decontamination of doorknobs.

**Results:**

When comparing ATP values on the same type of doorknobs, significant differences in contamination were demonstrated among several clinical rooms and several rooms in wards during all three seasons. No correlation was observed between ATP values on clinical-examination-room doorknobs and outpatient numbers, or between ATP values at any monitoring point and microbial colony-forming units. ATP values on clinical-examination-room doorknobs were reduced after cleaning according to instructions.

**Conclusions:**

ATP assay is useful for measuring baseline doorknob contamination in clinical rooms. Our findings confirm the need to improve routine decontamination in clinical departments. We need to analyze further the relationship between hospital-acquired infections and doorknob contamination, as assessed by ATP assay in clinics.

## Background

The spread of healthcare-associated pathogens, such as methicillin-resistant *Staphylococcus aureus* and vancomycin-resistant *Enterococcus*, most frequently occurs through the transiently contaminated hands of healthcare providers [1]. Additionally, environmental contamination in clinics contributes to the transmission of healthcare-associated pathogens [2–4]. Hospital-acquired infections (HAIs) can develop via microbe-contaminated environmental surfaces that are frequently touched by hands, such as doorknobs, guardrails in corridors, and over-bed tables [5]. Thus, efforts to improve hand hygiene and isolation practices have routinely been implemented to prevent and control HAIs [5]. Healthcare providers also need to pay close attention to ensure adequate cleaning and disinfection of the hospital environment because these are significant parts of infection control programs [6].

Based on the concept of environmental cleaning and disinfection, recommendations and standards to improve hospital cleanliness have been published [7–9]. These guidelines recommend cleaning and disinfection of environmental surfaces in the proximity of patients, as well as surfaces that are likely to be touched by the patients, healthcare providers, or housekeepers. However, routine housekeeping practices are often suboptimal [10]. Therefore, increased attention should be paid to the effectiveness of cleaning protocols.

Approaches to monitoring the effectiveness of cleaning procedures include: (1) visual assessment of surfaces; (2) application of fluorescent dye to surfaces with subsequent assessment of residual dye after cleaning; (3) determination of aerobic colony-forming units (CFUs); and (4) detection of ATP on surfaces [11]. Detection of ATP, which is present in all types of organic material, including bacteria, food, and human secretions and excretions, on environmental surfaces, is used in the food and beverage industries to evaluate the adequacy of cleaning protocols [12]. The ATP bioluminescence assay as an indicator of general organic contamination [11, 13, 14] and the standard stamp agar method for monitoring microbiological contamination [14, 15] are both available to monitor hospital contamination. However, few investigators have used these methods to monitor doorknob contamination in medical settings, especially clinical departments.

Doorknobs are inevitable points of hand contact in domestic environments. In medical settings, there is a variety of doorknobs, including grip-, lever-, pull-, push-, and insert-types. Few studies have compared ATP levels on doorknobs surfaces during different monitoring periods. In general, well-cleaned surfaces with little organic material have an ATP yield <250–300 relative light units (RLUs), while poorly cleaned surfaces with a large amount of organic material can have >1,000 RLUs [11]. In the present study, we determined seasonal variations in contamination of various types of doorknobs in a university-affiliated hospital, using the ATP bioluminescence assay.

## Methods

### Determination of doorknob types and monitoring points

**Fig. 1:**
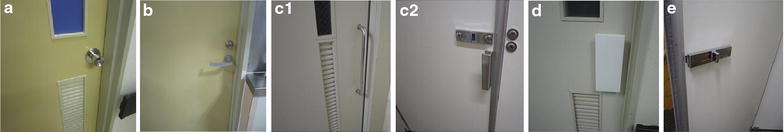
Grip-type (**a**), lever-type (**b**), two pull-types (**c1**, **c2**), push-type (**d**), and insert-type (**e**) of doorknobs. They were selected by all members of infection control team.

Before starting the monitoring of doorknob contamination, we determined the types of doorknobs to be assessed (i.e., grip-, lever-, pull-, push-, and insert-types) and the monitoring points to be evaluated (e.g., inpatient wards and outpatient clinics) in our 372-bed tertiary-care hospital. We prepared a list of all the doorknob types in the hospital: grip-type (a), lever-type (b), two pull-types (c1, c2), push-type (d), and insert-type (e) (Fig. 1), and then each member of the infection control team (ICT) chose a single type of doorknob. We selected the monitoring points to compare the level of contamination on different doorknob types in two different healthcare settings, inpatient wards and outpatient clinics. We chose 16 monitoring points in each of the wards and clinics (Table 1). There were no inaccessible locations because the sampling spots were limited and did not include clean rooms or patient treatment rooms. We did not explain the determination of monitoring points to the corresponding healthcare providers and housekeepers before starting this investigation, to avoid a monitoring effect on cleaning and to comprehend baseline data.

**Table 1:** ATP values and microbial colony-forming unit on several doorknob types in wards and clinics in different seasons

**Position in hospital**	**Monitoring point of doorknob**	**Type of doorknobs**	**Mean** ± **SD of ATP values (RLU)**	**Range of ATP values (RLU)**	**Microbial CFU per 10 cm** ^**2**^
<October to November, 2013>					
3rd floor in southern building	Staff lavatory	A**	404 ± 125	230–618	0
	Staff break room	B*	551 ± 251	250–860	1
4th floor in southern building	Staff lavatory	A**	334 ± 207	157–756	1
	Staff break room	B*	310 ± 204	107–740	8
	Linen closet	A	794 ± 308	421–1342	2
4th floor in southern building	Staff lavatory	A**	249 ± 161	101- 544	1
	Staff break room	B*	417 ± 112	217–567	0
5th floor in southern building	Staff lavatory	A**	365 ± 147	171- 642	3
2nd floor in northern building	Dirty utility room	C1	512 ± 232	212–821	3
	Staff break room	C1	429 ± 194	144–788	2
3rd floor in northern building	Dirty utility room	C1	327 ± 193	129–614	4
	Staff break room	C1	451 ± 172	221–734	3
4th floor in northern building	Dirty utility room	C1	351 ± 108	156–463	4
	Staff break room	C1	290 ± 80	203–436	0
5th floor in northern building	Dirty utility room	C1	271 ± 159	127–534	3
	Newborn care unit	C1	216 ± 62	147–296	4
Clinic of internal medicine	Western-style lavatory for men	C2	2536 ± 744	1369–3490	7
	Western-style lavatory for men	E	927 ± 295	433–1443	22
	Japanese-style lavatory for men	C2	1176 ± 698	314–2511	11
	Japanese-style lavatory for men	E	923 ± 351	570–1515	25
	Western-style lavatory for women	C2	1323 ± 615	543–2291	12
	Western-style lavatory for women	E	364 ± 227	187–857	6
	Japanese-style lavatory for women	C2	2278 ± 2981	363–8886	3
	Japanese-style lavatory for women	E	607 ± 629	243–2004	7
	Examination room for general internal medicine	B*	1702 ± 455	1109–2355	4
	Examination room for nephrology	B*	2109 ± 1560	745–5434	30
	Examination room for gastroenterology	B*	10387 ± 8096	2745–26425	14
Department of radiology	Dressing room 1 for radiological tests	A**	668 ± 299	331–1244	1
	Dressing room 2 for radiological tests	A**	356 ± 92	214–529	0
	Dressing room 3 for radiological tests	A**	679 ± 146	513–953	0
Department for health examination	Lavatory for men	D	488 ± 157	218–705	4
	Lavatory for women	D	216 ± 59	119–284	2
<January to March, 2014>					
3rd floor in southern building	Staff lavatory	A**	360 ± 246	173–843	10
	Staff break room	B*	668 ± 321	314–1099	2
4th floor in southern building	Staff lavatory	A**	4393 ± 6443	166–14174	8
	Staff break room	B*	719 ± 793	144–2125	5
	Linen closet	A	598 ± 171	327–829	0
4th floor in southern building	Staff lavatory	A**	203 ± 87	104–345	3
	Staff break room	B*	229 ± 49	200–327	10
5th floor in southern building	Staff lavatory	A**	444 ± 290	278–1025	2
2nd floor in northern building	Dirty utility room	C1	1495 ± 2588	194–6759	0
	Staff break room	C1	455 ± 436	125–1284	1
3rd floor in northern building	Dirty utility room	C1	551 ± 295	271–1069	17
	Staff break room	C1	484 ± 212	238–721	0
4th floor in northern building	Dirty utility room	C1	358 ± 119	147–467	2
	Staff break room	C1	301 ± 185	153–654	1
5th floor in northern building	Dirty utility room	C1	541 ± 1056	32–2694	0
	Newborn care unit	C1	312 ± 136	131–492	0
Clinic of internal medicine	Western-style lavatory for men	C2	2841 ± 3132	1277–9221	20
	Western-style lavatory for men	E	1120 ± 347	804–1725	9
	Japanese-style lavatory for men	C2	1736 ± 1118	855–3674	63
	Japanese-style lavatory for men	E	770 ± 121	604–918	6
	Western-style lavatory for women	C2	58605 ± 136900	1466–338031	8
	Western-style lavatory for women	E	4607 ± 8044	247–20327	26
	Japanese-style lavatory for women	C2	1844 ± 1786	758–5225	6
	Japanese-style lavatory for women	E	432 ± 237	276- 905	6
	Examination room for general internal medicine	B*	2045 ± 1228	950–3943	13
	Examination room for nephrology	B*	2992 ± 1110	1825–4411	19
	Examination room for gastroenterology	B*	2076 ± 731	1499–3494	18
Department of radiology	Dressing room 1 for radiological tests	A**	911 ± 376	511–1412	5
	Dressing room 2 for radiological tests	A**	365 ± 214	205–792	6
	Dressing room 3 for radiological tests	A**	1959 ± 1982	497–5883	18
Department for health examination	Lavatory for men	D	498 ± 160	335–803	14
	Lavatory for women	D	522 ± 197	292–853	17
<June to July, 2014>					
3rd floor in southern building	Staff lavatory	A**	492 ± 171	301–783	5
	Staff break room	B*	1277 ± 516	635–2178	2
4th floor in southern building	Staff lavatory	A**	310 ± 48	235–372	1
	Staff break room	B*	526 ± 428	147–1342	6
	Linen closet	A	489 ± 141	327–652	0
4th floor in southern building	Staff lavatory	A**	272 ± 117	134- 488	1
	Staff break room	B*	195 ± 78	70–291	9
5th floor in southern building	Staff lavatory	A**	498 ± 378	108–992	0
2nd floor in northern building	Dirty utility room	C1	997 ± 523	415–1615	6
	Staff break room	C1	578 ± 307	79–876	0
3rd floor in northern building	Dirty utility room	C1	694 ± 441	416–1566	8
	Staff break room	C1	452 ± 171	335–795	8
4th floor in northern building	Dirty utility room	C1	601 ± 206	343–892	0
	Staff break room	C1	772 ± 681	271–2104	3
5th floor in northern building	Dirty utility room	C1	233 ± 108	126–411	0
	Newborn care unit	C1	276 ± 90	137–401	1
Clinic of internal medicine	Western-style lavatory for men	C2	1681 ± 811	907–3095	3
	Western-style lavatory for men	E	1307 ± 568	809–2388	1
	Japanese-style lavatory for men	C2	916 ± 353	500–1541	31
	Japanese-style lavatory for men	E	527 ± 226	84–710	12
	Western-style lavatory for women	C2	1454 ± 1549	531–4583	4
	Western-style lavatory for women	E	684 ± 549	191–1624	39
	Japanese-style lavatory for women	C2	989 ± 283	665–1306	7
	Japanese-style lavatory for women	E	415 ± 171	303–759	9
	Examination room for general internal medicine	B*	1938 ± 622	1333–2917	13
	Examination room for nephrology	B*	2428 ± 622	1464–3353	81
	Examination room for gastroenterology	B*	2312 ± 978	745–3410	116
Department of radiology	Dressing room 1 for radiological tests	A**	1165 ± 594	585–2187	61
	Dressing room 2 for radiological tests	A**	572 ± 262	254–943	5
	Dressing room 3 for radiological tests	A**	773 ± 208	559–1156	3
Department for health examination	Lavatory for men	D	999 ± 635	498–2176	0
	Lavatory for women	D	1386 ± 2177	261–5807	2

Type of doorknobs included grip-type (A), lever-type (B), two pull-types (C1 and C2), push-type (D), and insert-type (E).

*SD* standard deviation, *RLU* relative light unit, *CFU* colony-forming unit.

* When comparing ATP values on lever-type doorknobs, there was a significant difference in contamination between the clinical examination rooms and staff break rooms in the wards.

** There was a significant difference in ATP values on grip-type doorknobs of dressing rooms for radiological tests, as compared with the same type of doorknobs of staff lavatories in the wards.

### Frequency and season of monitoring of doorknob contamination

We performed the monitoring between 17:00 and 18:00 h on every Tuesday for 7 weeks in three different seasons. The ATP bioluminescence assay and standard stamp agar method were carried out six times and once out of seven times during the same monitoring period, respectively. Our main assessment was of contamination with organic material (bacteria, food, and human secretions and excretions) using the ATP assay, and the stamp agar method was used in a confirmatory manner for bacterial isolation. The monitoring seasons were: October to November (autumn) 2013, January to March (winter) 2014, and June to July (summer) 2014 because incidences of bacterial gastroenteritis and food poisoning were presumed to have seasonal variation. The same ICT members performed the sampling and assessment with the ATP assay and stamp agar culture. Outpatients alone touched the doorknobs of the clinical examination rooms, and the number of visiting outpatients was estimated to be equal to the frequency of touch of the doorknobs. We also analyzed the correlation between ATP values on the clinical doorknobs and the number of outpatients.

### ATP bioluminescence assay

This assay was done using a Lumitester PD-20 (Kikkoman Biochemifa, Tokyo, Japan). We selected this device because it was one of most popular devices in Japan. The samples were collected by wiping a 3.5 × 3.5 cm area (roughly equivalent to the area of a stamp agar plate) of each doorknob with a specialized swab provided by the assay (LuciPac Pen), and the ATP values of the swabs were measured immediately. The same ICT member collected the samples from each area throughout this study, to avoid wiping bias. We also measured the ATP values on an uncontaminated grip-type doorknob (six times). This type was chosen because the grip-type was only available as an experimental model.

### Standard stamp agar method

A commercially available stamp agar plate (DD Checker Soybean Casein Digest Agar Plate; Kyokuto Pharmaceutical Industrial Co., Ltd., Tokyo, Japan) was used to monitor microbial contamination on each doorknob (10-cm^2^ area roughly equivalent to the ATP wiping area). The stamp agar method was also performed by the same ICT member who did the wiping throughout the study, to avoid bias in wiping and stamping. The stamped agar plates were immediately cultured for 24–72 h under aerobic conditions with moisture at 35°C, and colonies were counted. The data were estimated as CFU per 10 cm^2^. We also identified microorganisms to the genus or species level. Correlations between ATP values and microbial counts on the doorknobs were analyzed.

### Feedback of contamination data to healthcare providers and housekeepers

After completing the monitoring, we provided the contamination data to the appropriate healthcare providers and housekeepers. The time of cleaning, disinfectants used, and how to clean the environment were investigated in detail through the interview with the staff and inspection of the environment when cleaning. If high ATP values were found, we instructed the relevant individuals on optimal cleaning methods using the established manual. In such cases, we assessed whether the ATP values were reduced, using measurement by the same ICT member using the same sampling method at 17:00–18:00 h 1 week after instruction.

### Ethical considerations

The study protocol was approved by the ethical committee of Kitasato Medical Center before starting the investigation. During the sample and data collection, we protected the privacy and confidentiality of the personal information, under supervision of the hospital manager.

### Statistical analysis

Data collected from all of the samples were transferred to the program Excel Add-in for statistical analysis. The data concerning RLU were expressed as the mean ± standard deviation and range (minimum to maximum). When comparing the ATP values on the same type of doorknob at different monitoring points during the same study period, the data were analyzed using the Mann–Whitney *U* test, as previously described [11]. Spearman’s correlation index γ was calculated using statistical analysis software. A *p* value of <0.05 was considered to be statistically significant.

## Results

### ATP values and microbial CFU on several doorknob types in wards and clinics in different seasons

ATP values on uncontaminated grip-type doorknobs using the simulation were 24 ± 11 (12–41) RLU, which were the negative control levels. The relationship between ATP values and microbial CFU per 10 cm^2^ of several doorknob types is shown for the three different seasons (Table 1).

When comparing ATP values on lever-type (b) doorknobs, there was a significant difference in contamination between the clinical examination rooms and staff break rooms in the wards during each season (average RLU; 4,733 vs. 426 in autumn, 2,371 vs. 413 in winter, and 2,270 vs. 666 in summer). In addition, there was a significant difference in ATP values on grip-type (a) doorknobs of dressing rooms for radiological tests, as compared with the same type of doorknobs of staff lavatories in the wards during each of the three seasons. In contrast, there was no significant difference in ATP values on pull-type (c1) doorknobs of dirty utility rooms in the wards and the same type of doorknobs of staff break rooms during each of the three seasons.

### Identification of microbes in wards and clinics by type of doorknobs

The types and numbers of microorganisms in wards and clinics according to type of doorknob are shown in Table 2. When it was impossible to identify the microbial genus or species, we described them as non-fermentative rods, Gram-positive rods, Gram-positive cocci, and fungi. The main types of microbes attached to the various doorknobs were coagulase-negative *Staphylococcus*, *Bacillus*, *Corynebacterium*, and *Micrococcus* in both wards and clinics.

**Table 2:** Cumulative data of microbes in wards and clinics by type of doorknobs

Type of doorknobs	Genus- or species-level identification of organisms in wards (CFU/10 cm^2^ for each)	Genus- or species-level identification of organisms in clinics (CFU/10 cm^2^ for each)
A	CNS (10), *Corynebacterium* (10), *Bacillus* (6), *Micrococcus* (6), NFR (3), *Aspergillus* sp (1), GPR (1)	CNS (80), *Micrococcus* (16), *Corynebacterium* (1), NFR (1), GPR (1)
B	CNS (27), *Bacillus* (9), *Micrococcus* (6), NFR (1)	CNS (227), *Bacillus* (73), GPR (5), *Micrococcus* (1), *B*. *cereus* (1), *Corynebacterium* (1)
C1	CNS (33), *Micrococcus* (15), *Bacillus* (13), *Corynebacterium* (3), GPR (3), NFR (2), Fungi (1)	NT
C2	NT	CNS (130), *Bacillus* (34), *Micrococcus* (8), *Corynebacterium* (3)
D	NT	*Bacillus* (15), CNS (13), GPR and GPC (5), *Micrococcus* (5), *Acinetobacter* (1)
E	NT	CNS (93), *Bacillus* (31), *Corynebacterium* (29), *Micrococcus* (11), GPR (2), NFR (1), *S*. *aureus* (1)

Type of doorknobs included grip-type (A), lever-type (B), two pull-types (C1 and C2), push-type (D), and insert-type (E).

*CFU* colony-forming unit, *CNS* coagulase-negative staphylococcus, *NFR* non-fermentative rods, *GPR* gram-positive rods, *GPC* gram-positive cocci, *NT* not tested.

### Correlation between ATP values on clinical booth doorknobs and outpatient numbers

**Fig. 2:**
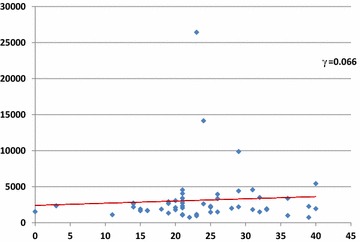
Correlation between ATP values on clinic booth doorknobs and outpatient numbers were determined. The X-axis, Y-axis, and γ indicate the outpatient numbers, ATP values, and correlation coefficient, respectively.

We determined the correlation between ATP values on clinical booth doorknobs and outpatient numbers in the three seasons (Fig. 2). We found no strong correlation (correlation index γ = 0.066).

### Correlation between ATP values and microbial CFU on doorknobs

**Fig. 3:**
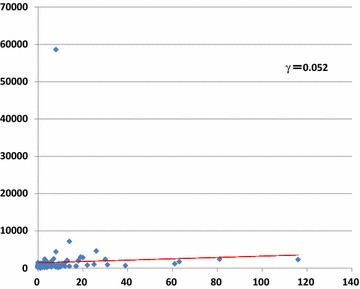
Correlation between mean ATP values of each monitoring point and microbial CFU per 10 cm^2^ were determined on doorknobs. The X-axis, Y-axis, and γ indicate the CFU, ATP values, and correlation coefficient, respectively.

We determined the correlation between mean ATP values of each monitoring point and microbial CFU per 10 cm^2^ on doorknobs in the three seasonal periods (Fig. 3). We observed no strong correlation (correlation index γ = 0.052).

### Feedback of contamination data to healthcare providers and housekeepers

After completion of monitoring, we provided feedback in the form of the collected contamination data to the relevant healthcare providers and housekeepers. The time of cleaning was 07:00–10:00 h at every point in the wards and clinics, and 19:00–20:00 h in the clinical examination rooms (general internal medicine, nephrology, and gastroenterology). Quaternary ammonium compounds were used for environmental disinfection. High mean ATP values >1,000 RLU were found in the clinical examination rooms and adjacent lavatories with lever-type (b) and pull-type (c2) doorknobs during all study seasons (Table 1). Therefore, we instructed the housekeepers on the recommended cleaning methods. One week after this instruction, the ATP values on doorknobs after cleaning were reduced in the clinical examination rooms (*n* = 3) and adjacent lavatories (*n* = 4) (693, 229, 408, 731, 431, 274, and 175 RLU, respectively). The second ATP value after instruction was 420 ± 203 (175–731) RLU, while the first value before instruction was 5,024 ± 30,044 (314–338,031) RLU (Table 1).

## Discussion

During all three seasons, there were significant differences in contamination among several types of clinical rooms (e.g., examination rooms and dressing rooms for radiological tests), and among several types of rooms on the wards (e.g., staff break rooms and lavatories), when comparing ATP levels on the same type of doorknob. We found no correlation between ATP levels on clinical-examination-room doorknobs and outpatient numbers, or between ATP levels at any monitoring point and microbial counts. The high ATP levels on clinical-examination-room doorknobs were decreased by cleaning after intervention by the ICT.

Omidbakhsh et al. [16] recently reported a limitation of the ATP assay for assessing decontamination of environmental surfaces in healthcare settings. Although the ATP method demonstrated acceptable linearity and repeatability, most of the disinfectant chemicals quenched or enhanced the ATP values variably in different settings. However, in the present study, there might have been less potential interference with disinfectant compounds, because the interval between the latest cleaning and ATP measurement was 7–21 h. When assessing contamination by ATP values, it is necessary to confirm the latest time of cleaning by disinfectants.

Watanabe et al. [14] studied hospital cleanliness in three Japanese hospitals that were specialized in long-term care. Based on ATP values and CFU per stamp medium, they showed that nursing stations, including the doorknobs, were cleaner than patient areas and public spaces, including the guardrail in the corridor. Our observations also indicated that nursing areas in wards were cleaner than public areas in clinics. Furthermore, there was no significant correlation between ATP values and numbers of inpatients in the previous study [14]. Similarly, we found no strong correlation between ATP values on doorknobs in clinical examination rooms and the numbers of outpatients.

Gastroenteritis (61 cases a year) associated with contamination of environmental surfaces was reported as an HAI in an old and architecturally unsatisfactory children’s hospital [17]. In our study, we selected different monitoring periods because the incidences of bacterial gastroenteritis and food poisoning in the clinics and wards were presumed to show seasonal variation. There was a significant difference in contamination assessed by ATP values between the clinical examination rooms and staff-break rooms in the wards during each season. Thus, contamination monitoring needs to be performed regardless of the season. The main microorganisms found on the various doorknobs were coagulase-negative *Staphylococcus*, *Bacillus*, *Corynebacterium*, and *Micrococcus* in the wards and clinics. Appropriate disinfectants should be selected according to the data for microbial isolation.

It is likely that there has been little evaluation of the adequacy of routine housekeeping practices in some medical settings [11]. Previous investigations showed that cleaning of patient care areas was often suboptimal and that environmental surfaces appeared to remain contaminated with microorganisms after routine cleaning [10, 18–20]. Based on the ATP values in our study, there was a significant difference in contamination between the clinical examination rooms and staff break rooms in the wards. We provided feedback in the form of the collected contamination data to the relevant housekeepers, and instructed them on the recommended cleaning methods. Although visual inspection of the cleaned surfaces is assumed to be adequate, the surfaces that meet visual criteria for cleanliness often remain contaminated with pathogens or other organic material [21]. Therefore, quantitative approaches including the ATP method are warranted for adequate evaluation of the effectiveness of cleaning practices [11, 21]. Huang et al. [22] compared visual inspection, aerobic CFU, and ATP assay to evaluate surface cleanliness at a medical center. The ATP assay was a sensitive and rapid tool for evaluating the quality of cleaning. In our study, after providing the housekeepers with relevant cleaning instructions, cleaning of doorknobs resulted in reduced ATP values in the clinical examination rooms and adjacent lavatories, although there was a limitation regarding a small number of second ATP values after cleaning instruction. When evaluating novel cleaning practices, baseline decontamination (i.e., the level of decontamination routinely achieved using normal cleaning procedures) must also be taken into consideration [13]. In this context, it is worth noting a recently reported assessment of daily cleaning practices using the ATP method in a hospital in a developing country [23]. This study suggests that visual assessment is not enough to ensure quality of the process and it is necessary to document the level of cleanliness by quantitative methods. However, ATP value should not be interpreted as a surrogate indicator for the presence of microbial pathogens [24].

## Conclusion

We used an ATP assay to assess contamination of doorknobs in a hospital environment during different seasons. Doorknobs may become contaminated by frequent hand touching, resulting in HAIs. Comparison of ATP values on doorknobs showed significant differences in contamination among several types of clinical rooms and several types of rooms in wards during each of the three seasons. Our findings support the need for improvement of routine decontamination practice in clinical departments. We need to analyze further the relationship between HAI rates and doorknob contamination, assessed by ATP assay in clinics.

## Abbreviations

ATP: adenosine triphosphate
CFU: colony-forming unit
HAIs: hospital-acquired infections
ICT: infection control team
RLUs: relative light units
